# Short-term adaptation to sound statistics is unimpaired in developmental dyslexia

**DOI:** 10.1371/journal.pone.0198146

**Published:** 2018-06-07

**Authors:** Yafit Gabay, Lori L. Holt

**Affiliations:** 1 Department of Special Education, University of Haifa, Haifa, Israel; 2 Edmond J. Safra Brain Research Center for the Study of Learning Disabilities, University of Haifa, Haifa, Israel; 3 Carnegie Mellon University, Department of Psychology, Pittsburgh, United States of America; Universidad de Salamanca, SPAIN

## Abstract

Developmental dyslexia is presumed to arise from phonological impairments. Accordingly, people with dyslexia show speech perception deficits taken as indication of impoverished phonological representations. However, the nature of speech perception deficits in those with dyslexia remains elusive. Specifically, there is no agreement as to whether speech perception deficits arise from speech-specific processing impairments, or from general auditory impairments that might be either specific to temporal processing or more general. Recent studies show that general auditory referents such as *Long Term Average Spectrum* (LTAS, the distribution of acoustic energy across the duration of a sound sequence) affect speech perception. Here we examine the impact of preceding target sounds’ LTAS on phoneme categorization to assess the nature of putative general auditory impairments associated with dyslexia. Dyslexic and typical listeners categorized speech targets varying perceptually from /ga/-/da/ preceded by speech and nonspeech tone contexts varying. Results revealed a spectrally contrastive influence of the preceding context LTAS on speech categorization, with a larger magnitude effect for nonspeech compared to speech precursors. Importantly, there was no difference in the presence or magnitude of the effects across dyslexia and control groups. These results demonstrate an aspect of general auditory processing that is spared in dyslexia, available to support phonemic processing when speech is presented in context.

## Introduction

Developmental dyslexia is a specific developmental disorder in learning to read that is not a direct result of impairments in general intelligence, gross neurological deficits, uncorrected visual or auditory problems, emotional disturbances or inadequate schooling [1]. Typical symptoms include poor phonological awareness, impaired verbal short term memory, and impaired lexical retrieval [2]. In line with this profile, a major guiding hypothesis has been that dyslexia involves a core phonological deficit in the access to, and manipulation of, phonemic language units [3, 4].

Nonetheless, there remains considerable debate about whether impairments in dyslexia are restricted to speech or whether they may reflect more general impairments [5–7]. Research directed at resolving this debate has focused largely on potential deficits in auditory temporal processing of rapidly-evolving sounds [8], in forming perceptual anchors against which incoming acoustic information may be compared [9, 10], or in a general capacity to establish short-term representations of sound stimulus statistics perhaps as a result of diminished repetition-induced adaptation [11, 12]. At the same time, other research suggests that there may be domain-general impairments in procedural learning among individuals with dyslexia that provide a basis for phonological deficits [13–15]. Recent work has indicated impairments in tracking probabilistic information across speech, nonspeech auditory, and visual perceptual input [16, 17] as well as procedural learning inefficiencies in auditory and visual domains [18, 19]. Despite empirical progress, there remains little consensus regarding the basis of the ubiquitous phonological deficits observed in dyslexia [20].

In this regard, a paradigm that has been widely studied among typical adults [21–25] and typically-developing children [26] is interesting in that it taps into phonological processing, as well as general auditory perceptual processes and sensitivity to the accumulation of probabilistic acoustic information across time. In these studies, listeners hear a speech syllable preceded by a probabilistic sequence of nonspeech sine-wave tones sampled from one of two distributions of tones varying in the spectral mean (i.e., higher- or lower-frequency distributions of tones). The resulting nonspeech precursor sequences sound something like a simple tune. When these tone sequences precede speech targets drawn from a series of syllables varying perceptually from /ga/ to /da/, tones sampled probabilistically from a higher-frequency distribution result in more /ga/ responses, whereas the same speech targets are more often categorized as /da/ when preceding tones are sampled from a lower-frequency distribution [21–23].

The influence of the preceding nonspeech precursors is *spectrally contrastive*. Sequences of tones with a lower-frequency spectral mean shift speech categorization toward response alternatives with greater high-frequency spectral energy whereas higher-frequency tone sequences shift categorization toward the speech category characterized by lower-frequency energy. As an example, /ga/ and /da/ are differentiated in large part by the onset frequency of the third formant (F3), which is lower for /ga/ and higher for /da/. Perceptually-ambiguous speech syllables, with F3 onset frequencies intermediate /ga/ and /da/ are more often perceived as /ga/ (the lower-frequency alternative) when preceded by a higher-frequency tone sequence. The same syllables are more often categorized as /da/ when lower-frequency tones precede it [21–23, 27]. Similar effects of preceding distributions of tones are observed for categorization of vowels [25] as well as Mandarin tone [24]. Speech categorization among typically-developing 5-year-olds is also influenced in the same manner [26]. In each case, the effects are *spectrally contrastive*, with regard to the spectral energy that differentiates speech categories.

Laing, Liu, Lotto, and Holt [21] point out that this constellation of findings is particularly interesting because the pattern of context dependence across these nonspeech-speech stimuli looks very much like classic demonstrations of talker normalization. A classic example of talker-dependent speech categorization was offered by Ladefoged and Broadbent *[28]*, who presented listeners with target words varying in the vowel within a /b_t/ frame at the end of a context phrase, *Please say what this word is*. Using early speech synthesis techniques, Ladefoged and Broadbent manipulated the context phrase by raising or lowering the first (F1) and/or second (F2) formant frequencies of the precursor phrases, conceptually modeling an increase or decrease in vocal tract length and, correspondingly, a change in talker. When phrases modeling different ‘talkers’ preceded the target words, vowel categorization in /b_t/ context shifted as a function of the ‘voice’ of the context phrase. For example, when a phrase consistent with a shorter vocal tract preceded the target, listeners reported hearing *bit* whereas they reported the same vowel to be *bet* when it was preceded by a sentence modeling a longer vocal tract. These results have long been interpreted to suggest that listeners extract some type of talker-specific information from context to ‘normalize’ speech perception, inasmuch as perception may compensate for vocal tract differences evident across talkers. In light of the influence nonspeech tone sequences have upon subsequent speech categorization [21], it is possible that the information extracted from prior context that influences speech categorization need not be talker-specific, or even speech-specific information. Holt and colleagues [22, 23, 25] suggest that general auditory processes that track statistical distributions of energy across the frequency spectrum and shift subsequent perception contrastively in relation to these distributions could play a role in the context dependent speech perception that has been taken as evidence of talker normalization.

In understanding these context dependencies, it is useful to note that the nonspeech tone sequences utilized in the Holt [22] studies were composed of individual sine-wave tones randomly sampled from frequency distributions defined by a specific mean frequency on a trial-by-trial basis. Thus, each nonspeech precursor stimulus was unique, with contexts defined probabilistically. As a result, only the long-term average spectrum (LTAS, the distribution of acoustic energy across frequency for the entire duration of the tone sequence) differentiated the context conditions. The influence of these statistically-defined tone sequences on subsequent speech categorization suggests that listeners may keep a running estimate of the distributionally-defined LTAS across both speech and nonspeech sounds and encode subsequent sounds relative to, and contrastively with, these running averages [21, 22, 25]. Spectral contrast as a function of the LTAS may be an effective, domain-general process contributing to accommodation of talker differences across speech [21–24, 26, 29–33], including normalization of the sort described by Ladefoged and Broadbent [28], accommodation of individual differences in overall voice pitch that impact Mandarin lexical tone realization [24], the ability to adapt to a speaker’s style [casual vs. careful; 32] and the inability to adapt to some particular voice changes [34].

In sum, there is evidence that the LTAS of incoming sounds, whether speech or nonspeech, impacts subsequent phonological processing among both typical adult and typically-developing child listeners. In this way the LTAS of probabilistically-defined preceding sounds appears to act as a referent for perception. Notably, the evidence indicates that these effects arise from general auditory processing, not specific to speech. In light of observations that individuals with dyslexia have phonological processing impairments [2], difficulty forming perceptual anchors [9], inefficiencies in learning across probabilistic information [16, 17] and dysfunction in forming short-term representations across sound statistics [11, 12], these characteristics make context-dependent speech categorization across precursors varying in LTAS a potentially useful tool for examining the nature of impairment in dyslexia.

The processing demands this paradigm places on auditory and speech processing make it possible that speech processing may not be impacted by context sounds’ LTAS among individuals with developmental dyslexia. First, one common element across these effects is that LTAS acts as a referent; listeners perceive subsequent speech relative to, and contrastively with, the LTAS of the precursor sounds. To the extent that individuals with developmental dyslexia have a poor ability to form a perceptual anchor [35], there may be difficulty establishing LTAS as a referent. Second, listeners’ sensitivity to LTAS, as a distributional characteristic emerging across a sequence of sounds, appears to involve general auditory processing inasmuch as effects can be elicited by nonspeech, as well as speech, precursors. Although many prior studies have pursued general auditory origins for the phonological impairments typical of dyslexia, these studies have tended to focus on putative impairments in temporal processing [36] and have not yet investigated spectral processing demands of the sort involved in these context effects. One prior study reports that a single preceding tone affects phoneme categorization among typically-developing children as well as children with dyslexia [37]. However, the spectral contrast effects elicited by single tones [38] have a different time course than those elicited by probabilistic sequences of tones drawn from a spectral distribution [23] and so it is unclear if they rely on the same underlying mechanisms. Third, the nonspeech tone sequences that precede speech syllables are defined *probabilistically* in that they sample a particular distribution across the spectral input dimension. This involves a distributional regularity across the context sounds. Individuals with developmental dyslexia exhibit impairments in other forms of distributional learning across auditory visual and speech stimuli [16–18]. Likewise, dyslexia has been reported to involve impaired implicit use of sound statistics[11]. Thus, the extent to which individuals with dyslexia exhibit context-dependent speech categorization in the present paradigm may inform mechanisms that are involved in both the etiology of developmental dyslexia, and mechanisms that drive these effects within auditory processing, more generally. Fourth, by varying the probabilistic short-term acoustic context history across trials, the paradigm described above may require accumulation of probabilistic acoustic information over time. The ability to extract probabilistic information has been observed to be impaired among people with dyslexia [16, 17]. On the other hand, if participants with dyslexia are able of compute LTAS, this would demonstrate intact distribution-based general auditory processing that may positively influence speech processing when speech targets are presented in context, as they are in most natural listening environments.

This latter point connects with another motivation for testing context-dependent speech categorization among individuals with developmental dyslexia. Laboratory studies traditionally have documented auditory or phonological processing deficits in those with developmental dyslexia across categorization of isolated speech exemplars. This may underestimate the phonological processing in real-world listening environments if mechanisms for capitalizing on context to support phonological processing are intact among individuals with dyslexia. For example, in labeling a series of speech stimuli that morph from one phoneme to another (e.g., /ga/ to /da/), individuals with dyslexia tend to exhibit shallower labeling slopes indicative of a less sharp boundary between phonetic categories [8, 39–42]. Recent research with typical adults and typically-developing children [26] makes clear that measuring phonological processing in this standard way can underestimate speech categorization abilities because syllables are presented in isolation. Hufnagle, Thissen and Holt [26], for example, documented shallow categorization curves among typically-developing 5-year-olds when /ga/-/da/ syllables were presented for labeling in isolation. However, phoneme categorization was sharper when these same children heard the syllables in the context of sequences of tone precursor sounds varying in LTAS that elicited spectrally contrastive context effects. Inasmuch as natural speech syllables are rarely encountered in strict isolation, this is an important caveat that should be considered in relation to establishing the nature of phonological impairments among individuals with dyslexia. In the current study, we examine speech categorization in the context of preceding speech and nonspeech contexts differing in LTAS among adults with developmental dyslexia.

## Method

### Participants

Fourteen participants with developmental dyslexia and an equal number of control volunteers participated. Participants were native-English university students in Pittsburgh with no reported sensory or neurological deficits, including attention deficit hyperactivity disorder. All came from families of middle to high socioeconomic status. Diagnosis of a comorbid developmental learning disability served as an exclusion criterion. All individuals included in the Dyslexia group had a well-documented history of dyslexia. Specifically, (1) each individual had received a formal evaluation of dyslexia by a qualified psychologist; (2) each individual’s evaluation was verified by the diagnostic and therapeutic center at his or her university; and (3) each individual was receiving accommodations in educational settings. The Control group was age-matched with the Dyslexia group, with no diagnosed reading impairment and the same level of intelligence as measured by the Raven’s Standard Progressive Matrices (SPM) test [43]. All individuals included in the Control group had no history of learning disabilities and performance at or above average on standardized measures of reading. Written informed consent was obtained from all participants. The study was approved by Carnegie Mellon University Institutional Review Board (IRB) and it was conducted in accordance with the Declaration of Helsinki.

All participants performed a series of cognitive tests (see appendix 1 for a detailed description) to evaluate general cognitive ability (as measured by Raven’s Progressive Matrices; Raven, [43]), verbal working memory (as measured by the forward and backward Digit Span from the Wechsler Adult Intelligence Scale [44]; rapid automatized naming [45] and phonological awareness[46]. In addition, all participants performed both un-timed and timed (fluency) tests of word reading and decoding skills. In particular, participants performed the Word Identification (WI) and Word Attack (WA) subtests from the Woodcock Reading Mastery Test-Revised; WRMT-R [47] and they also performed the Sight Word Efficiency, Forms A+B (i.e., rate of word identification) and Phonemic Decoding Efficiency, Forms A+B (i.e., rate of decoding pseudo-words) subtests from the Test of Word Reading Efficiency; TOWRE-II[48].

As indicated by results shown in Table 1, the groups did not differ in age or cognitive ability. However, compared to the Control group, the Dyslexia group exhibited a profile of reading disability conforming to the symptomatology of developmental dyslexia. This group differed significantly from the Control group on word reading and decoding skills in both rate and accuracy measures (Table 1). In addition, the Dyslexia group showed characteristic deficits in the three major phonological domains: phonological awareness (Spoonerisms), verbal short-term memory (digit span) and rapid naming (rapid automatized naming).

**Table 1:** Demographic and psychometric data of dyslexia and control groups.

	Group					
Measure	*Dyslexia* *Mean (SD)*	*Range*	*Control* *Mean (SD)*	*Range*	*P*	Cohen’s d
Age (in years)	20.78 (3.21)	18–30	21.5 (2.73)	18–29	.*57*	.23
Raven’s SPM	56.42 (2.79)	51–60	57.85 (1.95)	54–60	.*12*	.59
Digit spanª (combined)	10.5 (2.47)	7–16	13.64 (3.07)	6–18	.*01*	1.12
RAN objectsª	106.14 (18.68)	74–129	118.64 (13.46)	93–133	.*05*	1.1
RAN colorsª	100 (13.67)	80–120	111.14 (7.82)	97–124	.*05*	.76
RAN numbersª	103.78 (12.95)	63–113	114.57 (3.41)	109–120	.*01*	1.13
RAN lettersª	103.16 (6.35)	85–111	114.57 (6.93)	105–117	.*01*	1.68
WRMT-R WIª	99.42 (5.57)	92–113	116.50 (6.83)	100–126	.*01*	1.79
WRMT-R WAª	96.78 (7.83)	87–115	116.5 (13.35)	100–137	.*01*	1.8
TOWRE SA (A+B)ª	97.78 (8.55)	81–112	117.28 (6.82)	101–127	.*01*	2.51
TOWRE PD (A+B)ª	91.42 (7.83)	72–112	113.57 (13.35)	100–127	.*01*	2.36
Spoonerism time	126.58 (52.98)	13–224	91.5 (30.21)	63–156	.*05*	.81
Spoonerism accuracy	8.21 (3.35)	1–12	11.14 (2.10)	4–12	.*01*	1.04

ªStandard scores (whereby smaller numbers are expected for dyslexia group), other scores are raw scores. Raven scores are presented in percentiles.

Note that all participants in the Dyslexia group were high functioning university students with dyslexia. Prior studies of dyslexia have revealed that such participants achieve average performance on standardized reading tests (including tests that involve low-frequency words such as word identification from the Woodcock Reading Mastery Test-Revised), but nevertheless vary significantly from matched control groups and continue to present phonological problems that can be assessed by phonological tests such as the Spoonerism test [49]. Participants in the Dyslexia group fit this profile. The Dyslexia group differed significantly from the Control group across all literacy measures and exhibited phonological processing impairments (as indicted by the Spoonerism test), despite average performance on standardized tests. This profile is typical of a sample of dyslexic adults.

### Stimuli

#### Speech targets

**Fig 1:**
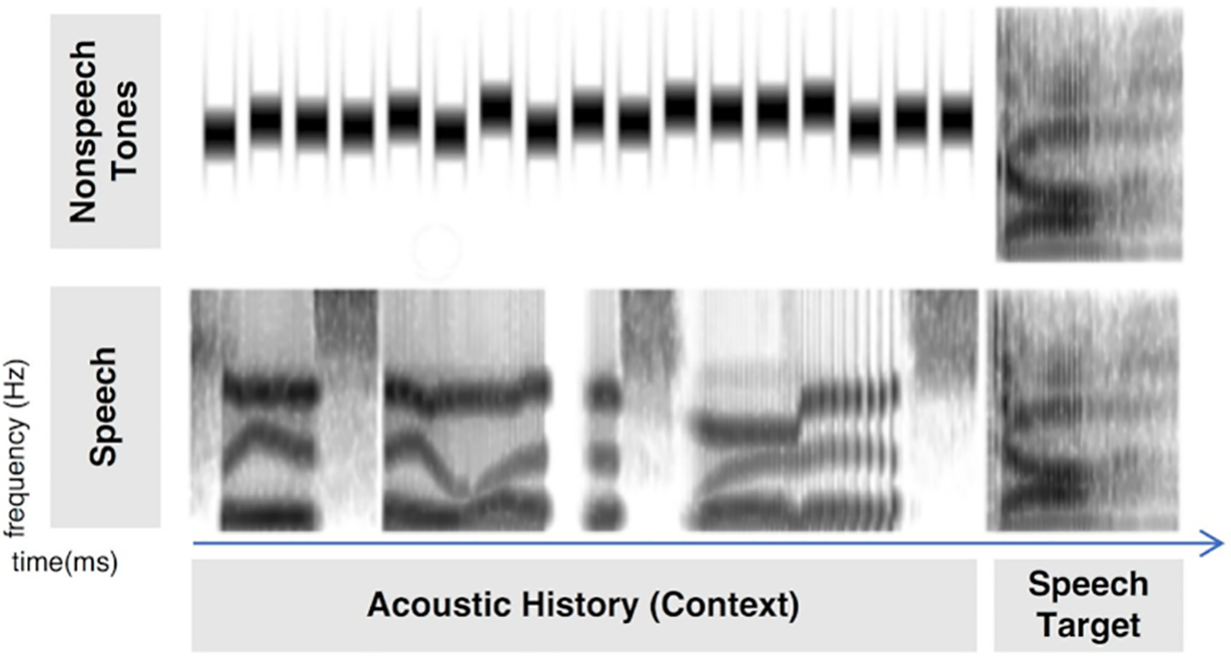
A schematic illustration of stimulus construction. The top panel shows a spectrogram (time x frequency) of a single nonspeech tone context stimulus with a High LTAS preceding a perceptually unambiguous /ga/ syllable. The bottom panel shows the High LTAS speech context (*Please say what this word is…*) preceding a perceptually unambiguous /da/.

Nine speech target stimuli were derived from natural /ga/ and /da/ recordings from a monolingual male native English speaker (Computer Speech Laboratory, Kay Elemetrics, Lincoln Park, NJ, USA; 20-kHz sampling rate, 16-bit resolution) and were identical to those utilized in several earlier studies [22, 23, 50]. To create the nine-step series, multiple natural productions of the syllables were recorded and, from this set, one /ga/ and one /da/ token were selected that were nearly identical in spectral and temporal properties except for the onset frequencies of F2 and F3. Linear predictive coding (LPC) analysis was performed on each of the tokens to determine a series of filters that spanned these endpoints (Analysis-Synthesis Laboratory, Kay Elemetrics) such that the onset frequencies of F2 and, primarily, F3 varied approximately linearly between /ga/ and /da/ endpoints. These filters were excited by the LPC residual of the original /ga/ production to create an acoustic series spanning the natural /ga/ and /da/ endpoints in approximately equal steps. Creating stimuli in this way provides the advantage of very natural-sounding speech tokens. These 411-ms speech series members served as categorization targets. Fig 1 illustrates the stimuli. Notice that the main difference between the targets is the onset frequency of the third formant (F3) in the range of approximately 1800–2800 Hz. Likewise, the concentration of acoustic energy in the LTAS of the speech and nonspeech targets differs in this spectral region. Each speech target was RMS matched in energy to the /da/ endpoint.

#### Speech context stimuli

Following the approach of Laing, Liu, Lotto, and Holt [21] and building from the work of Ladefoged and Broadbent [28], two speech contexts were synthesized. Formant frequencies and bandwidths from a recording of a male native English speaker reciting ‘*Please say what this word is*…’ were extracted and used as parameters to synthesize the phrase using the parallel branch of the Klatt synthesizer [51]. From these baseline synthesis parameters, third formant (F3) center frequency and bandwidth parameters were manipulated to create two “talkers.” One “talker” was synthesized to possess relatively higher-frequency energy in the F3 region with a peak in energy at about 2866 Hz. Another “talker” was created with relatively lower-frequency F3 energy peaking at about 1886Hz. These manipulations resulted a Context LTAS (High, Low) independent variable across speech contexts. Pairing each of these two 1700-ms contexts with the nine speech targets (50-ms inter-stimulus interval) resulted in 18 unique stimuli. These stimuli were mixed across High and Low Context Frequency and randomized for presentation. Twenty such randomized blocks resulted in a total of 360 speech context trials. Speech stimuli were sampled at 11.025 kHz, and matched in RMS energy to the speech targets.

#### Nonspeech context stimuli

Following the methods of previous studies [21, 23], the LTAS differences between the High and Low speech contexts in the F3 region were modeled with two distributions of sine-wave tones to create nonspeech contexts that varied in their LTAS. Whereas the LTAS of the speech contexts inherently possesses energy across the frequency spectrum, the nonspeech contexts explicitly sample acoustic energy only in the third formant (F3) frequency region of the spectrum significant to /ga/-/da/ categorization by sampling sine-wave tones within a limited frequency band. Thus, with nonspeech contexts, it is possible to focus acoustic energy precisely on the spectral regions predicted by prior studies of spectral contrast [23, 52] to have an effect on target /ga/-/da/ categorization, specifically energy in the region of F3.

These sequences of tones were similar to those described by Holt (23). They did not sound like speech and did not possess articulatory or talker-specific information. Seventeen 70-ms tones (5 ms linear onset/offset amplitude ramps) with 30 ms silent intervals created 1700-ms nonspeech contexts matched in duration to the speech contexts. As in previous experiments [21–23, 30], the order of the tones making up the nonspeech contexts was randomized on a trial-by-trial basis to minimize effects elicited by any particular tone ordering. Thus, any influence of the nonspeech contexts on the speech categorization is indicative of listeners’ sensitivity to the LTAS of the context and not merely to the simple acoustic characteristics of any particular segment of the tone sequence. It should be noted that the final tone of the sequence was constant. This prevented any differences between conditions from arising from the impact of the tone temporally adjacent to the speech targets. The frequency of the final tone was 2300 Hz, intermediate between the distribution means defining the High and Low LTAS tone contexts.

As in Laing, Liu, Lotto, and Holt [21], the bandwidth of frequency variation of the distributions from which nonspeech tones were sampled to create the tone sequence contexts was approximately matched to the bandwidth of the peak in the corresponding speech contexts’ LTAS, as measured 10 dB below the peak. The low-frequency F3 distribution sampled 435 Hz in 29 Hz steps around a distribution mean (1873.5 Hz, range 1656–2091 Hz) that modeled the F3-energy of the Low LTAS speech context. The high-frequency F3 distribution sampled 570 Hz in 38 Hz steps around a mean (2785 Hz, range 2500–3070Hz) that modeled the F3-energy of the High LTAS speech contexts. Tones from these High and Low frequency distributions were randomly ordered to create 360 unique contexts, with 20 High frequency sequences and 20 Low frequency sequences. The final, constant 2300 Hz tone was appended to each and, a 50-ms silent interval separated the 17-tone sequences from the speech target. Tones comprising the nonspeech contexts were sampled at 11.025 kHz and matched in energy to the speech targets.

### Procedure

Listeners categorized the nine speech targets in each of the four contexts (speech/nonspeech x high/low LTAS). Speech and Nonspeech contexts were presented in separate blocks, with High and Low LTAS contexts mixed with each block. The order of blocks was counterbalanced across participants. Within a block, trial order was random. On each trial, listeners heard a context plus speech target stimulus and categorized the speech target as /ga/ or /da/ using buttons on a computer keyboard corresponding to labels on a monitor mounted in front of participants.

The two categorization blocks were followed by a brief discrimination test to measure the extent to which manipulations of the LTAS were successful in producing perceived talker differences across the High and Low speech contexts. On each trial, participants heard a pair of context sentences and judged whether the voice speaking the sentences was the same or different by pressing buttons on a computer keyboard. The task was divided into two blocks, with a brief break between blocks. Within a block, listeners heard both the High and Low speech context stimuli across 20 randomly-ordered trials. One-half of the trials were different talker pairs (High-Low or Low-High, five repetitions each) and the remaining trials were identical voices (High-High, Low-Low, five repetitions each).

For both speech categorization and talker discrimination tests, acoustic presentation was under the control of E-Prime [53] and stimuli were presented diotically over linear headphones (Beyer DT-150) at approximately 70 dB SPL (A) with participants seated in a sound-attenuating booth. The experiment lasted approximately an hour.

## Results

**Fig 2:**
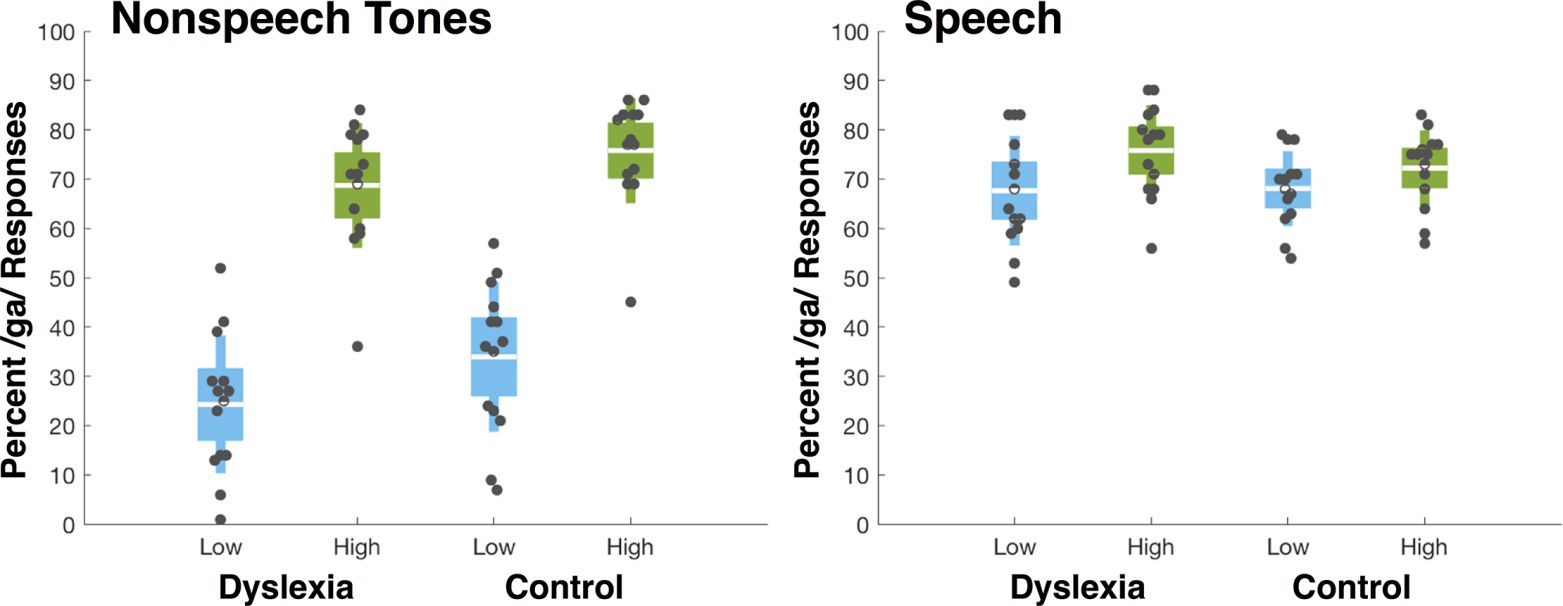
Mean percent /ga/ responses as a function of context type (speech, nonspeech tone), context LTAS (High, Low) and group (dyslexia, control). Dots represent individual participant’s data. Each box shows the mean (white line) and 95% confidence intervals for the mean. Blue boxes correspond to Low LTAS contexts whereas green boxes illustrate High LTAS contexts. Thus, the spectrally contrastive influence of context is evident as greater /ga/ responses for High (green) compared to Low (blue) LTAS contexts.

Fig 2 plots the results. Following the approach of Laing, Liu, Lotto, and Holt [21] we conducted an analysis of variance (ANOVA) with context type (Speech vs. Nonspeech), context LTAS (High vs. Low) and speech target (/ga/ vs. /da/) as within-subjects factors and group (Dyslexia vs. Controls) as a between subjects factor with percent of /ga/ responses as the dependent variable. The context type (Speech vs. Nonspeech) main effect was significant *F* (1, 26) = 66.253, *p* = .001, *η*_*p*_^*2*^ *=* .71, indicating that listeners more often reported the speech targets to be /ga/ following speech, compared to nonspeech, contexts. This likely arises from the necessary spectral differences between the speech and nonspeech contexts, since speech is a wideband signal and the nonspeech tone sequences sample a limited spectral range. There was also a significant main effect of speech target, *F*(8, 208) = 315.0, *p* = .001, *η*_*p*_^*2*^ *=* .92, indicating that /ga/ responses varied as intended across the speech targets.

Additionally, there was a robust main effect of context LTAS (High, Low) on speech target categorization, *F*(1, 26) = 237.75, *p* = .001, *η*_*p*_^*2*^ *=* .901. This influence was consistent with patterns of spectral contrast observed in prior research [54]. When the preceding phrase or nonspeech tone sequence had greater acoustic energy in higher frequencies in the F3 frequency band, listeners more often categorized the following target as /ga/ (M = .73, SE = .015), compared to categorization of the same target following the phrase or nonspeech tone sequence sampling lower F3 frequencies (M = .48, SE = .016). The context type by group interaction was marginally significant, *F* (1, 26) = 3.9, *p* = .06, *η*_*p*_^*2*^ *=* .12. Further analysis revealed that the nonspeech contexts elicited more /ga/ responses among the Control, compared to the Dyslexia group, *F* (1, 26) = 4.1, *p* = .053 whereas there was no difference across groups for speech contexts, *F*<1. The target by group interaction was not significant indicative of similar identification curves between the two groups, *F* (8, 208) = 1.3, *p* = .24, *η*_*p*_^*2*^ *=* .04.

Of most interest to the present research question, the context frequency by group interaction was not significant, *F* (1, 26) = 1.1, *p* = .3077, *η*_*p*_^*2*^ *=* .04. The LTAS of preceding speech and nonspeech contexts affected phoneme categorization just as much among individuals in the Dyslexia group as those in the Control group. The three-way interaction of context type, context frequency, and group was not significant, *F*<1, indicating that the magnitude of influence of speech and nonspeech precursors’ LTAS was consistent across the Dyslexia and Control groups.

Higher order interactions involving speech targets were also significant (*p* < .01). However, since our predictions center on context-dependent speech target categorization, the focus of interpretation is placed on interactions that do not involve target.

## General discussion

The present study examined the influence of the long-term average spectrum (LTAS) of preceding speech and nonspeech contexts on speech categorization among typical and dyslexic listeners. Prior research with typical young adult [22–24, 54, 55]and child listeners [26] demonstrates that the LTAS of preceding sound, whether speech or nonspeech, affects speech categorization in a spectrally-contrastive manner [54, 55]. Sounds with a greater concentration of high-frequency energy push speech categorization toward lower-frequency alternatives whereas contexts with greater lower-frequency energy shift categorization to higher-frequency alternatives. Single preceding speech syllables [56] and nonspeech sinewave tones [52] also influence speech categorization in a spectrally contrastive manner, including among children with dyslexia [37]. However, the influence of probabilistic acoustic energy evolving across sentence-length utterances or nonspeech sequences has a distinct time course from the spectral contrast effects evoked by single precursor tones, suggesting the possibility that different mechanisms contribute [23, 38].

We hypothesized that the processing demands of tracking LTAS across acoustic contexts may present difficulties for listeners with dyslexia for several reasons. First, sensitivity to the LTAS may relate to the ability to form a perceptual anchor inasmuch as listeners categorize subsequent speech targets relative to, and contrastively with, the LTAS of precursor sounds. If listeners with developmental dyslexia are impaired in their ability to form a perceptual anchor [9], they may have difficulty establishing the LTAS of precursor sounds as a ‘perceptual anchor’ against which to inform speech categorization. This possibility is all the more intriguing because LTAS-dependent spectral contrast effects, like perceptual anchor effects [11, 12, 57], have been hypothesized to arise from neural adaptation [22]. Second, since most studies of general auditory processing in dyslexia have concentrated on temporal processing [8, 36], there is quite little information to inform an understanding of how spectral-domain processing demands impact speech categorization in dyslexia. Third, the probabilistic nonspeech tone contexts used in prior studies with typical adult listeners have involved extracting information across probabilistic, distributional regularities in sound input [23]. Since individuals with dyslexia can exhibit impaired processing of statistical regularities present across sounds [16] and reduced sensitivity to probabilistic information [17], the statistically-defined, probabilistic LTAS of nonspeech precursor sounds may not influence speech categorization as it does in typical listeners. Lastly, we sought to examine the possibility that intact context-dependent speech categorization in dyslexia may provide unexpected support for speech categorization in real-world environments, compared to the contextually-impoverished listening conditions in which phoneme perception is typically assessed in the laboratory.

Our results replicate previous findings with typical adult and child listeners [21–26]. In particular, we observe that the LTAS of speech and nonspeech precursor sounds influences speech categorization among typical listeners in a spectrally contrastive manner. When participants heard contexts with greater low-frequency acoustic energy, categorization was shifted toward the syllable with greater high-frequency energy, /da/. The same syllables were more often reported as /ga/ (characterized by greater low-frequency energy) when contexts had greater higher-frequency acoustic energy. Notably, there was a larger magnitude LTAS effect for nonspeech compared to speech precursors, consistent with previous observations [54]. This likely arises from the concentrated energy present in a targeted spectral band in nonspeech contexts compared to the more distributed spectral information necessarily present in speech.

Most critically, we observed that individuals with dyslexia also exhibit spectrally contrastive, context-dependent speech categorization. In fact, the influence of distributionally-defined probabilistic nonspeech tone contexts and sentence-length speech contexts was equivalent to the influence observed for typical listeners. This indicates that listeners with dyslexia track evolving spectral statistics from sound and use it to influence phonetic perception in the manner of typical listeners.

In prior research, Holt [22] has found that the task employed in the current investigation demands that listeners are sensitive to the spectral mean of the distribution of probabilistic nonspeech tone precursors. This is especially interesting with regard to the possibility that individuals with dyslexia have difficulty in forming a perceptual anchor, described also as a reduced sensitivity to sound regularities [58] that may be related to impairments in neural adaptation [57] or faster decay of implicit memory across sound statistics [11]. This perspective has been mostly gained support from studies revealing that individuals with dyslexia are less able to benefit from simple item repetition in the context of frequency discrimination and speech-in-noise tasks [58]. Recently these observations have been extended to much more complex regularities embedded in richer stimulus statistics. Typical listeners are capable of extracting summary statistics across longer sequences of sounds [59]. People with dyslexia, however, are less able to use this information. That is, their perception is less biased toward the experienced mean and they tend to exhibit smaller bias towards mean frequency embedded in a stimuli in the context of auditory frequency discrimination [35] and visual spatial frequency discrimination tasks [57], consistent with the anchoring deficit hypothesis [9]. Compatibly, adults and children with dyslexia exhibit reduced neural adaptation across words, objects, faces and voice [12]. Notably, however, the pattern of results observed has not always been consistent; several studies have demonstrated that the ability to form a perceptual anchor is unimpaired among adults with dyslexia in both the visual [60] and auditory modalities [61, 62], including the language domain [63, 64]. The present results demonstrate that individuals with dyslexia are able to extract a spectral mean evolving across more than a second of sound and use it to ‘anchor’ how subsequent speech acoustics are categorized. For the nonspeech tone condition, this ‘anchor’ was defined by evolving sound statistics as stimuli varied trial-by-trial. Future research will be need to resolve this seeming disparity.

The present results are also informative in the context of evidence of an impaired ability to implicitly extract probabilistic information among those with dyslexia. In our previous research, we have reported that individuals with dyslexia are impaired in extracting probabilistic information in both the auditory [16] and visual modalities [17]. Despite these cross-modal results, the current results underscore that it is too strong to conclude that individuals with dyslexia have a general impairment in processing probabilistic information. The nonspeech tone sequences of the present study were defined probabilistically, yet their LTAS influenced subsequent speech categorization. Future research will need to focus on the detailed processing demands involved in various ‘probabilistic’ tasks, as well as different stimulus regularities that may be considered to be ‘probabilistic,’ to determine where differences between typical and dyslexic listeners emerge.

The present stimuli were modeled after Laing, Liu, Lotto, and Holt [21], with nonspeech tones defined probabilistically within a condition’s LTAS distribution. This matched the probabilistic nature of F3 variation in the speech contexts. Prior studies with typical listeners often have employed an additional control in order to assure that the probabilistic sequence of nonspeech tones, and not just the final tone temporally-adjacent to the speech target, produce the contrast effect [22, 23]. The present study cannot rule out the possibility that listeners with dyslexia may differ from typical listeners in the perceptual weighting of context information that accumulates over time. Thus, future studies in which distributional characteristics of nonspeech contexts [22] will be informative in addressing whether there may be more subtle differences in how listeners with dyslexia track statistical regularities evolving in the LTAS.

The present results also bring up the intriguing possibility that traditional approaches to measuring speech perception in the laboratory may underestimated phoneme categorization abilities among individuals with dyslexia (and typical listeners, for that matter). Presenting isolated phonemes or syllables to listeners for identification strips away context sounds that, as the present results demonstrate, can support categorization. Speech exemplars that are perceptually ambiguous in isolation can be perceptually exaggerated in a spectrally contrastive manner by surrounding sound context. In this way, the shallow speech identification curves often associated with individuals with dyslexia [65] would be expected to sharpen with informative contexts, such as those present in real-world listening environments, are made available. Although the present research did not directly test this hypothesis, this was the case among typical 5-year-old listeners who categorized the very same /ga/-/da/ syllables used here [26].

Speech categorization deficits in dyslexia have been attributed to an auditory processing deficit that affects both speech and nonspeech stimuli, and that is specific to temporal, but not spectral, acoustic information [7, 8, 66]. The present results are consistent with the view that some aspects of spectral processing across speech and nonspeech stimuli may be unimpaired in dyslexia. In this context, it is worth noting that typical listener’s exhibit contrastive context-dependent effects of temporal information on speech categorization, as well [67]. In a paradigm much like that of the present study, Wade and Holt found that preceding sequences of tones varying in their duration (and therefore rate of presentation) impacted how typical young adult listeners categorized /ba/-/wa/ syllables created to vary along a temporal formant-transition duration dimension. Future studies of temporal contrast in context-dependent speech categorization in dyslexia are likely to be informative. The present results demonstrate that ‘normalization’ of speech categorization as a function of the preceding long-term average spectrum of sound, whether speech or nonspeech, is intact in individuals with dyslexia and it is as efficient as it is among typical listeners.

## Appendix 1 –psychometric tests

The following tests were administered according to the test manual instructions:

1. *Raven’s Standard Progressive Matrices test* (Raven, Court & Raven, 1992)–Non-verbal intelligence was assessed by the Raven’s-SPM test. This task requires participants to choose an item from the bottom of the figure that would complete the pattern at the top. The maximum raw score is 60. Test reliability coefficient is .9
2. *Digit Span from the Wechsler Adult Intelligence Scale* (WAIS-III; Wechsler, 1997)—In this task, participants are required to recall digits presented auditorily in the order the were presented with a maximum total raw score 28. Task administration is discontinued after a failure to recall two trials with a similar length of digits. Test reliability coefficient is .9
3. *Rapid Automatized Naming* (Denkla, & Rudel, 1976)—The tasks require oral naming of rows of visually-presented exemplars drawn from a constant category (RAN colors, RAN categories, RAN numerals, and RAN letters). It requires not only the retrieval of a familiar phonological code for each stimulus, but also coordination of phonological and visual (color) or orthographic (alphanumeric) information quickly in time. The reliability coefficient of these tests ranging between .98 to .99.
4. *Woodcock Reading Mastery Test Word Identification and Word Attack subtests* (Woodcock & Johnson, 1990). The Word Identification subtest measures participants’ ability to accurately pronounce printed English words, ranging from high to low frequency of word occurrence with a maximum of total raw score 106. Test reliability coefficient is .97. The Word Attack subtest assesses participants’ ability to read pronounceable nonwords varying in complexity with a maximum total raw score of 45. Test reliability coefficient is .87. Task administration is discontinued when 6 consecutive words are read incorrectly.
5. *Sight Word Efficiency* (i.e., rate of word identification) and *Phonemic Decoding Efficiency*, (i.e., rate of decoding pseudowords) subtests from the Test of Word Reading Efficiency (TOWRE-II; [68]) were used to measure reading rate. The test contains two timed measures of real word reading and pseudo word decoding. Participants are required to read the words aloud as quickly and accurately as possible. The score reflects the total number of words/nonwords read correctly in a fixed 45-s interval. Task administration is discontinued after 45 seconds. Sight word efficiency maximum raw score is 108. Phonemic decoding efficiency maximum raw core is 65. Test-retest reliability coefficients for these subtests are .91 and .90 respectively.
6. *Spoonerism Test* (adapted from [69])—This test assesses the participants’ ability to segment single syllable words and then to synthesize the segments to provide new words. For example, the word pair “Basket Lemon” become “Lasket Bemon”. The maximum raw score is 12.
